# Design and Implementation of a Linear Active Disturbance Rejection Control-Based Position Servo Control System of an Electromotive Valve for Exhaust Gas Recirculation

**DOI:** 10.3390/s24051393

**Published:** 2024-02-21

**Authors:** Xin Cheng, Jianzhong Yin, Xiaokang Li, Rougang Zhou, Chong Fu

**Affiliations:** 1 School of Information Engineering, Wuhan University of Technology, Wuhan 430070, China; 2 School of Mechanical & Electronic Engineering, Wuhan University of Technology, Wuhan 430070, China; yjz252289@whut.edu.cn (J.Y.); lixiaokang0216@163.com (X.L.); fucong@whut.edu.cn (C.F.); 3 School of Mechanical Engineering, Hangzhou Dianzi University, Hangzhou 310000, China; 4 Wenzhou Institute, Hangzhou Dianzi University, Wenzhou 325013, China; 5 Mstar Technologies, Inc., Hangzhou 310012, China

**Keywords:** exhaust gas recirculation (EGR), electromotive valve, extended state observer (ESO), linear active disturbance rejection control (LADRC), position servo control system

## Abstract

An exhaust gas recirculation (EGR) valve is used to quickly and dynamically adjust the amount of recirculated exhaust gas, which is critical for improving engine fuel economy and reducing emissions. To address problems relating to the precise positioning of an electromotive (EM) valve under slowly varying plant dynamics and uncertain disturbances, we propose a servo control system design based on linear active disturbance rejection control (LADRC) for the EGR EM valve driven by a limited angle torque motor (LATM). By analyzing the structure of the LATM and the transmission, the dynamic model of the system is derived. In addition, to solve the problems caused by slowly varying plant dynamics and uncertain disturbances, we combine the effects of uncertain model parameters and external disturbances as the total disturbance, which is estimated in real time by an extended state observer (ESO) and then compensated. In addition, accurate angular information is obtained using a non-contact magnetic angle measurement method, and a high-speed digital communication channel is established to help implement a closed-loop position control system with improved responsiveness and accuracy. Simulation and experimental results show that the proposed servo system design can effectively ensure the precision and real-time performance of the EM valve under slowly changing plant dynamics and uncertain disturbances. The proposed servo system design achieves a full-stroke valve control accuracy of better than 0.05 mm and a full-stroke response time of less than 100 ms. The controlled valve also has good robustness under shock-type external disturbances and excellent airflow control capability. The repeatability of the airflow control is generally within 5%, and the standard deviation is less than 0.2 m^3^/h.

## 1. Introduction

Exhaust gas recirculation (EGR) technology, which recirculates a portion of the exhaust gas into the air intake pipe and back into the combustion chamber of the engine to participate in combustion, thereby optimizing the combustion environment and reducing NOx emissions, is widely used in both gasoline and diesel vehicles [1,2,3].

The EGR rate is defined as the ratio of the amount of exhaust gas recirculated to the total amount of intake air drawn into the cylinder [4]. In general, increasing the EGR rate can significantly reduce NOx emissions, but it is important to consider not only the effect on NOx emissions but also the energy efficiency of the engine when controlling the EGR rate. The specific requirements are as follows: (1) When the engine is running at full load, the exhaust gas mixed with fresh air will reduce the calorific value, and the combustion speed and combustion temperature will decrease, resulting in a decrease in the maximum power output of the engine. (2) When the engine is at medium load, the higher EGR rate will result in a higher fuel consumption rate. (3) When the engine is under a low load, especially when idle, the introduction of exhaust gas will cause unstable combustion or even lead to misfiring. Therefore, the EGR rate should be dynamically adjusted according to the temperature and load state of the engine to find the optimal solution between optimal engine performance and NOx emission [5,6,7,8]. Moreover, to reduce the complexity of the engine multi-parameter tuning process, the EGR valve’s own position servo control is decoupled from the exhaust gas intake demand control under the engine multi-parameter operating parameters. That is, the engine control system outputs the valve opening degree command, while the EGR position servo system completes the specific high-precision and real-time response position control.

There are uncertain disturbance problems in the practical application of the EGR valve, such as the shock of exhaust gas flow on the valve under different working conditions, and repeated switching of dynamic and static friction of the moving pairs, the vibration problems caused by the vehicle road surface, etc. The application conditions are complex, and the disturbances are difficult to model [9,10,11,12]. Another unfavorable situation is that due to the application, environmental factors such as carbon accumulation, pollution, and exhaust gas corrosion, can cause slow changes in the state of the springs and motion pairs, forming slow-varying system dynamics [13,14]. However, dynamic EGR rates require the exhaust gas intake to be precisely matched to the current engine combustion process. Otherwise, it will further lead to inadequate engine combustion, which, in turn, reduces engine efficiency and worsens emissions. Therefore, it is necessary to improve the robustness of the electromotive (EM) valve in the presence of the above uncertainties to achieve better dynamic response and valve control accuracy [15].

In response to the above problems, PID and its improvement methods are widely used for EGR valve position control. Choi et al. [16] used a three-loop cascade control method to achieve the position control of the EGR valve. H.J. Kim et al. [17] proposed a cascaded proportional–proportional integral (P–PI) controller based on a feedforward controller to implement the position control scheme of the EGR valve, which solved the problem of its poor response at lower control frequencies and compensated the output of the feedforward controller by the relationship between the controller output, speed command, and speed limiter. Bhuiyan et al. [18] proposed P–PI control with friction compensation for the phenomenon of position control oscillation when using PI control under mechanical systems with high differential friction. Ashok et al. [19] estimated unknown disturbed load torque using a friction observer in a model based on PID control. The study in [20] proposed a method for electronic throttle position control problem using a Drosophila optimization algorithm to tune the fuzzy PID control parameters.

Model predictive control and nonlinear internal mode control are modern control methods that are suitable for complex systems with dynamic characteristics. Rajaei et al. [21] proposed an EGR valve position servo control system based on model predictive control and experimentally proved that the full-open response time and the shut-off response time of the EGR valve are 500 ms and 200 ms, respectively. The study in [22] designed a nonlinear internal mode control strategy for linear motion valve position control to compensate for the effect of rate-dependent hysteresis. The study in [23] proposed on-line optimization position control strategies and conducted experimental and comparative studies on the dynamic response speed and control accuracy of EGR systems.

State observer is usually used to measure the system state that cannot be measured directly. Rajaei [24] proposed generalized predictive control to achieve closed-loop control of valve position. In order to control the valve more accurately, the torque spring is considered an external disturbance torque, and then the Kalman filter is used to estimate the torque value of the torque spring. The study in [25] estimated and suppressed the unmodeled dynamics and unknown disturbances in electromechanical throttle valves using a disturbance observer and designed the approach time-optimal controller to achieve high dynamic performance. The study in [26] proposed the use of a state observer to estimate the total disturbance of the system and designed an integral sliding mode controller with a dual closed loop of position and velocity loops to achieve the control of throttle openings and angular velocities.

Although PID and its improved methods have achieved better results in certain electronically controlled valve applications, there is a lack of effective evaluation methods for controlled objects whose model changes can lead to difficulties in tuning their control parameters. Modern control methods, such as model predictive control or internal mode control, have a high computational overhead and place high demands on the processor power of the control system. Due to the presence of components such as springs and the complex friction-switching process, the EM valve has highly nonlinear and slowly varying dynamics. In response to the above, we propose a design of the position servo control system of the EM valve for exhaust gas recirculation. The uncertainties of internal model mismatch and external disturbances are combined as the total disturbance, and an ESO is developed to estimate and compensate for this total disturbance to achieve a high level of robustness and accuracy.

The main contributions of the paper are highlighted as follows.

- A novel type of electromotive valve driven by LATM is introduced. We analyze the dynamics of the system through the models of LATM and transmission.
- For the EM valve application in exhaust gas recirculation, we propose a new approach to estimate and compensate for the uncertainties of both internal model mismatch and external disturbances.
- The parameters of the EM valve are obtained from the laboratory.
- The proposed approach introduces a disturbance rejection technique to ensure the accuracy and real-time performance of the EM valve under slowly varying plant dynamics and uncertain disturbances.

The rest of the paper is organized as follows. In Section 2, the model of the discussed EM valve is presented, and then in Section 3, the position servo controller based on LADRC is designed and verified through the use of simulation. The implementation of the position servo system is presented in Section 4, and the experimental verification is presented in Section 5. Finally, the conclusions are given in Section 6.

## 2. Structure and Model of EM EGR Valve

### 2.1. Structure of LTAM-Driven EM EGR Valve

**Figure 1:**
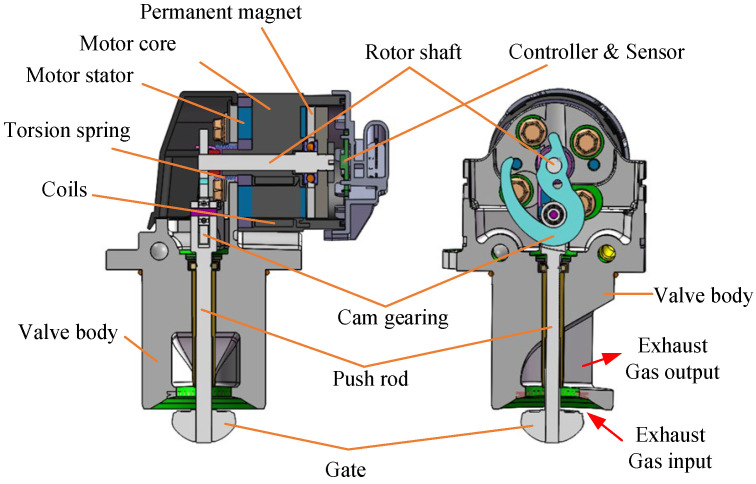
Structure of the electromotive EGR valve discussed.

The object discussed in this paper is an EM valve driven by a LATM motor, the structure of which is shown in Figure 1.

**Figure 2:**
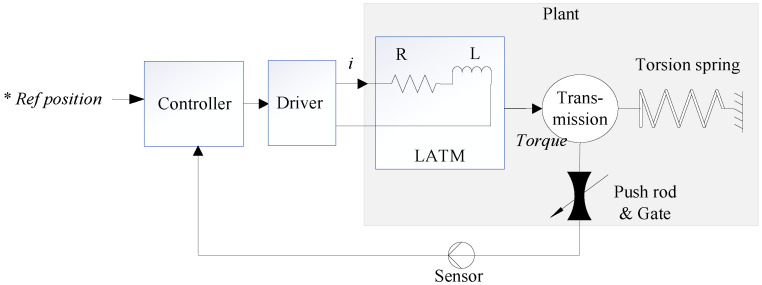
Block diagram of the electromotive EGR valve (*Ref represents desired value).

LATM can directly output torque to drive the load within a certain angular range, which has the advantages of large output torque, fast response, and high positioning accuracy [27,28]. The controller, driver, and sensor are integrated into the same PCB assembly. The sensor transmits the rotation angle information of the rotor shaft to the controller, which, in turn, generates a controlled current based on the *Ref position information. When the electromagnetic torque and torsion spring torque reach a state of equilibrium, a stable valve angle is established. In the initial state of the system, the torsion spring will ensure that the valve remains closed at all times. A brief system block diagram of the EGR valve structure is shown in Figure 2.

### 2.2. Plant Modeling

#### 2.2.1. Modeling LATM with Torsion Spring

LATM motors exhibit significant nonlinear characteristics, mainly in the nonlinearity of their EM torque and cogging torque [29]. However, a region with relatively ideal EM torque and cogging torque is selected based on simulation, in which the LATM is simplified into an ideal DC motor, and its torque equation is shown in Equation (1).

(1) $$T_{e}=K_{t}i_{a}$$

where $K_{t}$ represents the EM torque coefficient of the motor, and $i_{a}$ represents the current of the armature winding. We can obtain Equation (2).

(2) $$U_{i}=i_{a}R+L_{t}\frac{di_{a}}{dt}+E_{a}$$

where $U_{i}$ represents the armature voltage, R represents the equivalent resistance of the armature winding, $L_{t}$ represents the inductance of the motor winding, and $E_{a}$ represents the back EM force in Equation (3).

(3) $$E_{a}=K_{a}\omega$$

(4) $$\omega =\frac{d\theta }{dt}$$

where $K_{a}$ represents the back EMF coefficient, ω represents the angular speed of the motor, and *θ* represents the angular displacement of the shaft.

(5) $$U_{i}=d_{a}V_{in}$$

where $d_{a}$ represents the proportional coefficient related to the duty cycle of PWM, and $V_{in}$ represents the input voltage of the power stage. The motion equation is

(6) $$J_{e}\frac{d^{2}\theta }{dt^{2}}+f_{1}\frac{d\theta }{dt}=T_{e}-T_{k}-T_{h}$$

where $J_{e}$ represents the moment of inertia on the motor shaft, $f_{1}$ represents the viscous damping coefficient on the motor shaft, $T_{k}$ represents the spring torque, and $T_{h}$ represents the torque of the elastic shaft. The spring torque equation is shown in Equation (7).

(7) $$T_{k}=T_{0}+K_{x}\theta$$

where $T_{0}$ represents the initial torque of the torsion spring at the static position, and $K_{x}$ represents the torque coefficient of the spring. Based on Equations (1)–(7), the model of LATM is as shown in Figure 3.

#### 2.2.2. Modeling the Transmission Mechanism

The transmission adopts a force-locked eccentric cam through the torsion spring to make the eccentric cam maintain contact with the head of the valve stem in the process of movement, eliminating the gap between the valve stem head and the cam, whose installation position and spring characteristics must be estimated according to the torque force transmitted via the gear train [30]. The mechanical transmission equation of the LATM is shown in Equation (8).

(8) $$J_{a}\frac{d^{2}\theta_{a}}{dt^{2}}+f_{2}\frac{d\theta_{a}}{dt}=T_{s}-T_{2}$$

where $J_{a}$ represents the moment of inertia of the mechanical transmission, $\theta_{a}$ represents the turning angle of the eccentric cam, $T_{s}$ represents the torsional moment of the elastic shaft, $T_{2}$ represents the force moment such as air load, and $f_{2}$ represents the viscous friction coefficient. $T_{s}$ is obtained by electromagnetic torque minus friction in Equation (9).

(9) $$T_{s}=k(\theta -\theta_{a})$$

By combining Equations (1)–(7) and performing the Laplace transform, we can obtain

(10) $$\left\{\begin{array}{l} U_{i}(s)=RI_{a}(s)+L_{t}sI_{a}(s)+E_{a}(s)\\ E_{a}(s)=K_{a}s\theta (s)\\ J_{e}s^{2}\theta (s)+f_{1}s\theta (s)=T_{e}(s)-T_{0}-K_{x}\theta (s)-T_{h}\\ T_{e}(s)=K_{t}I_{a}(s) \end{array}\right.$$

By combining Equations (8) and (9) and performing the Laplace transform, we can obtain

(11) $$\left\{\begin{array}{l} J_{a}s^{2}\theta_{a}(s)+f_{2}s\theta_{a}(s)=T_{s}-T_{2}\\ T_{s}=k\left(\theta (s)-\theta_{a}(s)\right) \end{array}\right.$$

**Figure 4:**
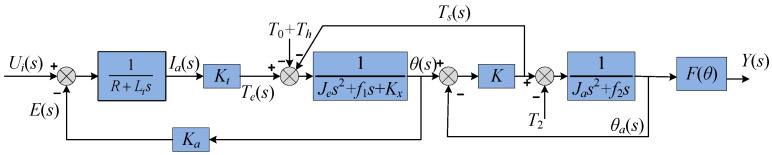
Block diagram of the plant model.

By combining Equations (10) and (11), we can obtain the plant model in Figure 4.

## 3. Linear ADRC Based Position Servo Control System

### 3.1. Overall Design of Position Controller Based on LADRC

**Figure 5:**
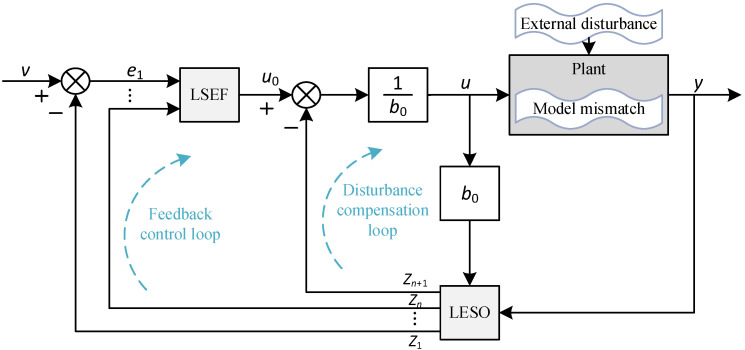
Block diagram of linear ADRC.

We adopted the method of ref. [31,32], using the observer bandwidth and controller bandwidth as intermediate variables to link the individual parameters, thus reducing the number of tuning parameters. The block diagram of the LADRC is shown in Figure 5, which consists of a linear extended state observer (LESO) and a linear state error feedback control law (LSEF) [33]. The tracking differentiator is not used because the differential transition process technique is more mature in engineering applications and to avoid the TD leading to phase lag and longer regulation time.

### 3.2. Order Analysis

The design of the LADRC must match the order of the controlled plant and the high-frequency gain value of the system shown in Figure 4, and we can obtain Equation (12).

(12) $$\left\{\begin{array}{l} U_{i}=i_{a}R+L_{t}\frac{di_{a}}{dt}+k_{a}\omega \\ J_{e}\frac{d^{2}\theta }{dt^{2}}=k_{t}i_{a}-T_{0}-k_{x}\theta -f_{1}\frac{d\theta }{dt}-T_{h}\\ J_{a}\frac{d^{2}\theta_{a}}{dt^{2}}=T_{s}-T_{2}-f_{2}\frac{d\theta_{a}}{dt}\\ T_{s}=K\left(\theta -\theta_{a}\right)\\ Y=F\left(\theta \right) \end{array}\right.$$

According to Equation (12), we have

(13) $$\left[\varepsilon_{1}s^{4}+\varepsilon_{2}s^{3}+\varepsilon_{3}s^{2}+\varepsilon_{4}s+K_{x}K\right]\theta_{a}=\frac{K_{t}K}{R+Ls}U-KT_{0}-\varepsilon_{5}T_{2}$$

where $\left\{\begin{array}{l} \varepsilon_{1}=J_{e}J_{a}\\ \varepsilon_{2}=J_{e}f_{2}+J_{a}f_{1}+\frac{K_{t}k_{a}J_{a}}{R+L_{t}s}\\ \varepsilon_{3}=KJ_{e}+k_{x}J_{a}+f_{1}f_{2}+KJ_{a}+\frac{K_{t}K_{a}f_{2}}{R+L_{t}s}\\ \varepsilon_{4}=K_{x}f_{2}+Kf_{1}+Kf_{2}+\frac{K_{t}K_{a}f_{2}}{R+L_{t}s}\\ \varepsilon_{5}=J_{e}s^{2}+K_{x}+f_{1}s+K+\frac{K_{t}K_{a}}{R+L_{t}s} \end{array}\right.$.

From Equation (13) and the parameters in Table 1, it can be known that the plant is a 4th-order model, as shown in Equation (14), as follows.

(14) $$\begin{array}{l} \left(0.0000000104s^{4}+0.0000019s^{3}+1.26s^{2}+181.2s+800\right)\theta_{a}=277.8U-\\ 5000T_{0}-\left(0.036s+0.0002s^{2}+5000.16\right)T_{2} \end{array}$$

where U is the power stage voltage, $T_{0}$ is the initial elastic torque, and $T_{2}$ is the torque equivalent to the external dynamic air disturbance on the camshaft.

From Equation (14), the coefficient of the 4th term in the denominator is eight orders of magnitude smaller than that of the 2nd term, and the coefficient of the 3rd term is six orders of magnitude smaller than that of the 2nd term. Due to the inherent switching characteristics of the power stage, it can be considered as both a proportional and a hysteresis object, and its transfer function is shown in Equation (15).

(15) $$G_{PWM}(s)=K_{PWM}\cdot e^{-T_{PWM}}$$

(16) $$e^{T_{PWM}}=1+T_{PWM}s+\frac{{\left(T_{PWM}s\right)}^{2}}{2!}+\frac{{\left(T_{PWM}s\right)}^{3}}{3!}+\cdots +\frac{{\left(T_{PWM}s\right)}^{n}}{n!}\approx 1+T_{PWM}s$$

Equation (15) can be equivalent to

(17) $$G_{PWM}\left(s\right)=K_{PWM}\cdot e^{-T_{PWM}}\approx \frac{K_{PWM}}{1+T_{PWM}s}$$

The frequency of the PWM signal is 20 kHz, $T_{PWM}=0.00005s$; therefore, Equation (17) can be simplified into Equation (18).

(18) $$G_{PWM}\left(s\right)\approx K_{PWM}$$

### 3.3. Design of LADRC

The plant model is expressed in Equation (19).

(19) $$\ddot{y}=f\left(y,\dot{y},d,t\right)+bu=-\lambda_{1}\dot{y}-\lambda_{0}y+d+b_{0}u$$

where $\lambda_{1}=\frac{\varepsilon_{4}}{\varepsilon_{3}}$, $\lambda_{0}=\frac{K_{x}K}{\varepsilon_{3}}$, $b_{0}=\frac{K_{t}KK_{PWM}}{R}$, $d=\left(-KT_{0}-\varepsilon_{5}T_{2}\right)\cdot K_{PWM}$, and y is the plant output. $f=-\lambda_{1}\dot{y}-\lambda_{0}y+d$ is regarded as the total disturbance, including unmodeled internal and external disturbances.

According to Equation (19), we select state variable $x_{1}=y$, $x_{2}=\dot{y}$, $x_{3}=f$, so the state space of the system is constructed in Equation (20).

(20) $$\left\{\begin{array}{l} {\dot{x}}_{1}=x_{2}\\ {\dot{x}}_{2}=x_{3}+b_{0}u\\ {\dot{x}}_{3}=\dot{f}\\ y=x_{1} \end{array}\right.$$

Rewrite Equation (20) to the matrix as follows

(21) $$\left\{\begin{array}{l} \dot{x}=Ax+Bu+E\dot{f}\\ y=Cx \end{array}\right.$$

where

(22) $$x=\left[\begin{array}{c} x_{1}\\ x_{2}\\ x_{3} \end{array}\right],A=\left[\begin{array}{ccc} 0 & 1 & 0\\ 0 & 0 & 1\\ 0 & 0 & 0 \end{array}\right],B=\left[\begin{array}{c} 0\\ b_{0}\\ 0 \end{array}\right],C=\left[\begin{array}{ccc} 1 & 0 & 0 \end{array}\right],E=\left[\begin{array}{c} 0\\ 0\\ 1 \end{array}\right]$$

The ESO is constructed in Equation (23).

(23) $$\left\{\begin{array}{l} {\dot{z}}_{1}=z_{2}+p_{1}\left(y-z_{1}\right)\\ {\dot{z}}_{2}=z_{3}+b_{0}u+p_{2}\left(y-z_{1}\right)\\ {\dot{z}}_{3}=p_{3}\left(y-z_{1}\right) \end{array}\right.$$

Rewrite Equation (23) as follows

(24) $$\left\{\begin{array}{l} \dot{z}=Az+Bu+L\left(y-Cz\right)\\ \hat{y}=Cz \end{array}\right.$$

where $z={\left[\begin{array}{ccc} z_{1} & z_{2} & z_{3} \end{array}\right]}^{T}$, $z_{1}$, $z_{2}$, $z_{3}$ are the observed estimates values of $x_{1}$, $x_{2}$, $x_{3}$, respectively, and $L={\left[\begin{array}{ccc} p_{1} & p_{2} & p_{3} \end{array}\right]}^{T}$ is the observer gain matrix.

Rewrite the following expression according to Equation (24)

(25) $$\dot{z}=\left(A-LC\right)z+Bu+Ly$$

The bandwidth method is used to set the LADRC parameters, and all the poles of the eigenequations in the LESO are configured to the left half of the real axis $-\omega_{0}$ in the s-plane by means of the pole configuration, which is to make the LESO effective. All eigenvalues of the A−LC are negative, as shown in Equation (26).

(26) $$\dot{z}=\left|sI-\left(A-LC\right)\right|=\left|\begin{array}{ccc} s+p_{1} & -1 & 0\\ p_{2} & s & -1\\ p_{3} & 0 & s \end{array}\right|={\left(s+\omega_{0}\right)}^{3}$$

(27) $$p_{1}=3\omega_{0},p_{2}=3\omega_{0}^{2},p_{3}=\omega_{0}^{3}$$

LSEF is calculated as

(28) $$u_{0}=k_{p}\left(y-z_{1}\right)-k_{d}z_{2}$$

where $u_{0}$ is the output control quantity after LSEF, and $k_{p}$ and $k_{d}$ are the proportional gain and differential gain of the controller, respectively. $k_{p}=\omega_{c}^{2}$, $k_{d}=2\xi \omega_{c}$, where $\omega_{c}$ is the bandwidth of the LSEF controller, ξ is the damping ratio.

LESO observes the disturbance $z_{3}$ in real time, and then compensates for it before the total disturbances shock the system.

The output of the controller is shown in Equation (29).

(29) $$u=\frac{u_{0}-z_{3}}{b_{0}}$$

**Figure 6:**
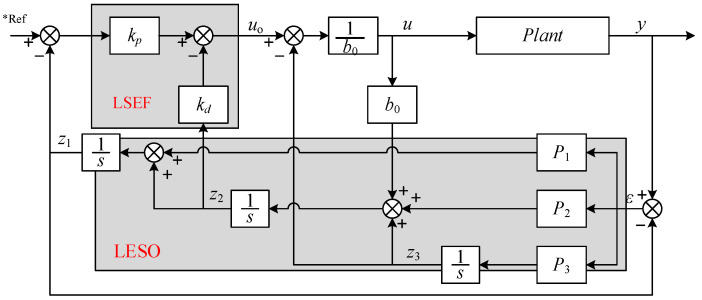
Block diagram of position closed-loop system based on LADRC (*Ref represents desired value).

Figure 6 shows the block diagram of a LADRC-based position closed-loop system for the EGR valve. After disturbance compensation, variable *u* acts with PLANT to form the desired *y.* Substituting Equation (29) into Equation (19), we have

(30) $$\ddot{y}=-a_{1}\dot{y}-a_{0}y+d+b_{0}u=f+b_{0}\frac{u_{0}-z_{3}}{b_{0}}=f+u_{0}-z_{3}\approx u_{0}$$

### 3.4. Parameter Tuning

By using the method of ref. [34], the LADRC parameters were obtained in Equation (31).

(31) $$P=\left(\begin{array}{c} p_{1}\\ p_{2}\\ p_{3} \end{array}\right)=\left(\begin{array}{c} 3\omega_{0}\\ 3\omega_{0}^{2}\\ \omega_{0}^{3} \end{array}\right)$$

(32) $$k_{p}=\omega_{c}^{2},\;k_{d}=2\omega_{c}$$

where $\omega_{0}$ is the observer bandwidth and $\omega_{c}$ is the controller bandwidth.

### 3.5. Simulation Verification

**Figure 7:**
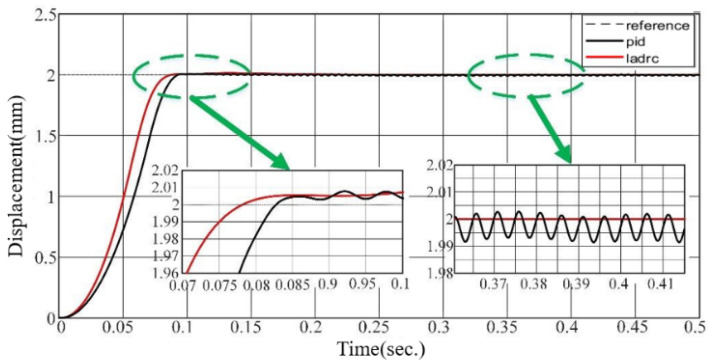
Step response performance of valve gate under LADRC and PID.

**Figure 8:**
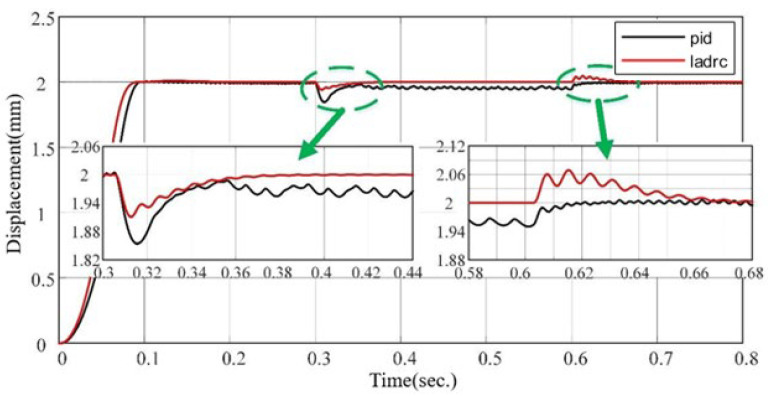
Comparison of disturbance rejection performance of LADRC and PID after an external disturbance.

**Figure 9:**
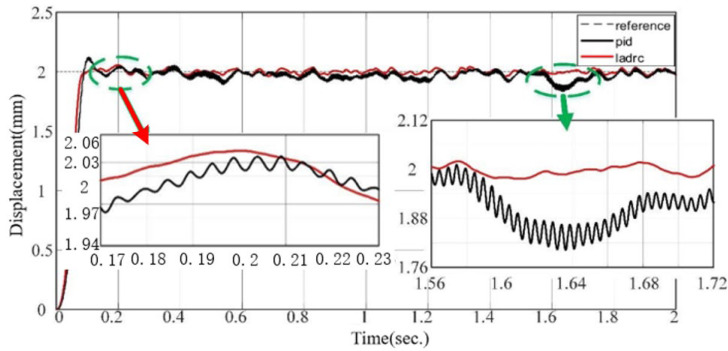
Comparison of disturbance rejection performance of LADRC and PID after the introduction of Gaussian white noise signal.

The performance of the LADRC is verified through comparison with the PID. In Figure 7, Figure 8 and Figure 9, we set the valve reference displacement to 2 mm so the reference displacement trajectory coincides with the 2 mm grid line in the figure.

It can be seen that the valve overshoot under LADRC control is smaller, its steady state error is resolved to zero, and the response time is significantly better than that of PID control. The reason is that the PID control is driven by the error, and the error is followed by the control variable output. In contrast, the method used in this paper can observe the unknown disturbance faster and, therefore, has a better real-time response.

Figure 8 shows that LADRC has better disturbance rejection performance when an external disturbance is applied at 0.3 s.

As shown in Figure 9, the valve gate displacement is set to 2 mm, and Gaussian white noise is used as the time-varying disturbance signal to simulate the continuous dynamic effect of vehicle road vibration, airflow, etc., on the valve disturbance, and the sampling time is set to 0.001 s. The overshoot of the PID after introducing the disturbance is 7%, and that of LADRC is less than 3%.

## 4. Design and Implement of Position Servo System

**Figure 10:**
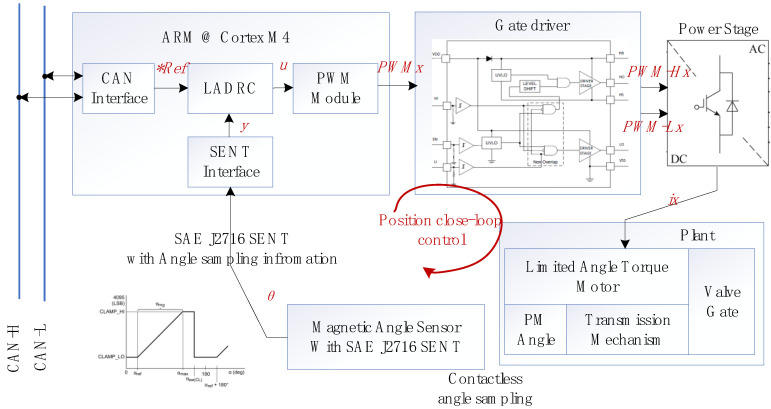
Block diagram of position servo system for EM valve (*Ref represents desired value).

A typical electromotive EGR valve is a CAN bus node controlled by the on-board ECU, which receives CAN bus commands to acquire the desired valve opening information. As shown in Figure 10, the position control system includes an ARM microcontroller, gate driver, power stage, controlled plant, and a non-contact magnetic angle sensor. A CAN bus interface obtains the reference position information *Ref of the valve from the top-layer ECU, while the SENT interface obtains the rotation angle information from the non-contact magnetic angle sensor and converts it into valve position according to the transmission model.

The LADRC (Figure 6) will complete the output of *u* to drive the power stage to generate the controlled current to form the electromagnetic torque of the LATM (Figure 3) and finally form the valve control. The EM valve servo control system has been successfully developed according to Figure 10 and applied to several samples, as shown in Section 5.

## 5. Experimental Verification

### 5.1. Gate Position Accuracy Verification under Slow-Varying Plant Dynamics

**Figure 11:**
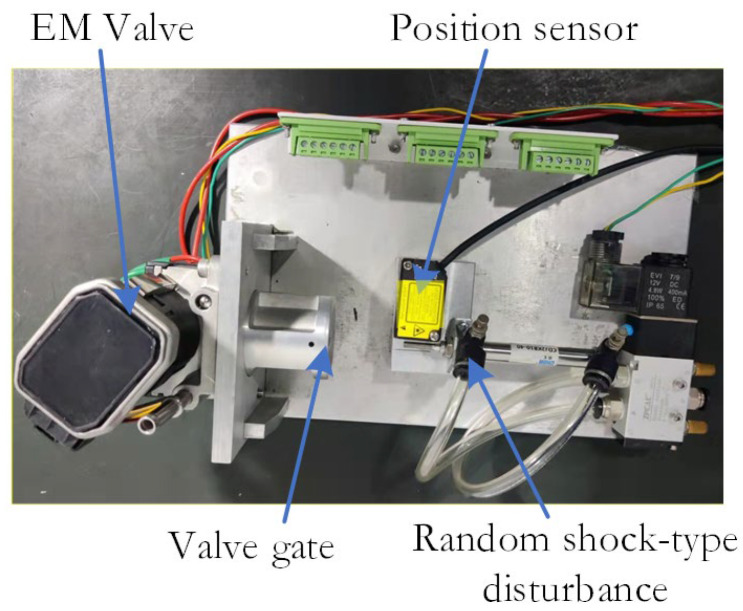
Experimental platform I.

The experimental platform I is shown in Figure 11, and the displacement sensor is Keens IL-S-065, which has a maximum sensing distance of 75 mm and a repeatable sensing accuracy of 2 μm. In addition, a cylinder (3.14 kN @ 0.4 MPa) is designed to provide a shock-type disturbance to the EM valve to verify the disturbance rejection performance of the proposed design.

**Figure 12:**
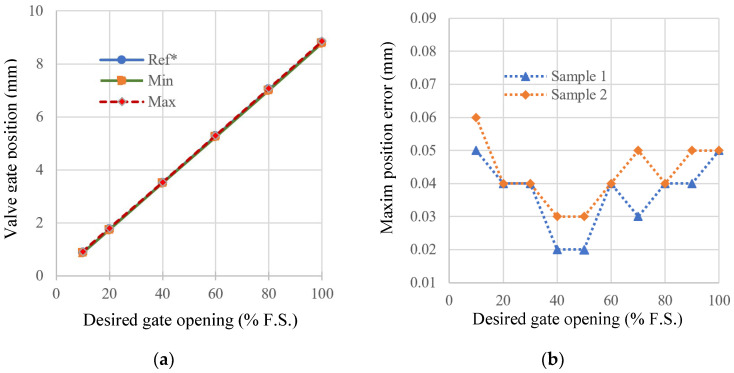
Valve gate position accuracy under different desired gate opening. (**a**) Position accuracy in ten groups of EGR valve samples. (**b**) Maximum position error of different samples.

Under identical conditions, linearity tests were performed on ten groups of EGR valve samples to determine the valve reference position as well as the positions corresponding to the minimum and maximum values, as shown in Figure 12. It is obvious that the design proposed in this paper has a high level of accuracy.

Sample 1 is the object with the parameters in Table 1. Sample 2 is another sample that was subjected to a 750 h life test with a modification to the motor that resulted in a 10% deviation in inductance and resistance, and the dynamic frictional force of its moving pair is 68 mN⋅m, which is 112.5% higher than that of Sample 1.

Valve positioning errors are shown in Figure 12b. Although Sample 1 shows slightly better accuracy compared to Sample 2, the overall positioning error remains at the same level. The results suggest that the approach used in this study has low sensitivity to slowly varying plant dynamics and effectively mitigates uncertainty in the internal model.

### 5.2. Step Response and Shock-Type Disturbance Rejection

**Figure 13:**
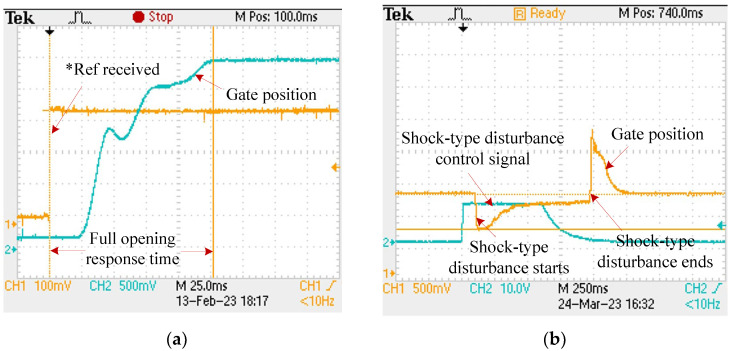
EM Valve performance verification. (**a**) Step response; (**b**) shock-type disturbance rejection (*Ref represents desired value).

Figure 13a shows that the step response time is 98 ms. However, the valve experiences a response delay of approximately 23 ms, which is influenced by factors such as CAN bus data parsing, LADRC computation overhead, and mechanical transmission delay. Figure 13b illustrates the ability of the EM valve to suppress shock-type disturbances. The EM valve has a maximum under-shock error of 1.07 mm and achieves a steady-state error of less than 0.001 mm.

### 5.3. Gas Flow Control Performance of EM Valve under Inner and External Uncertainties

**Figure 14:**
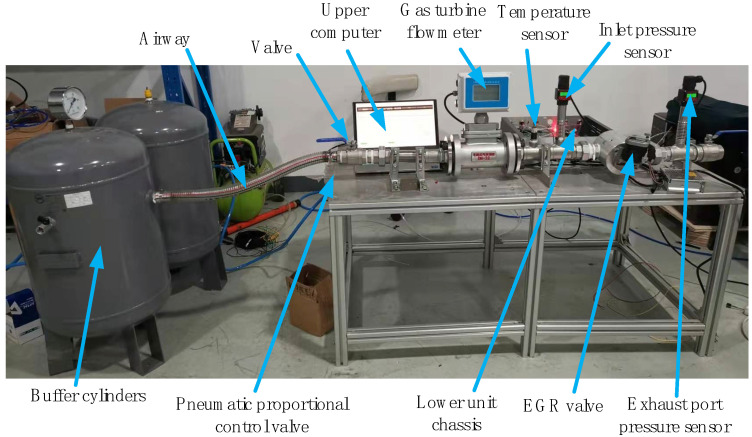
Experimental platform II.

Experimental platform II is shown in Figure 14. The airflow meter has a measurement accuracy of 0.1% F.S. and a maximum measurement range of 60 m^3^/h.

A flow meter was used to measure the airflow value of the pipeline. The sampling rate of the airflow is 100 Hz, and the valve opening is increased from 5% to 95% at 5% intervals to verify the relationship between valve openings and airflow and to compare with the theoretically fitted values at each opening point.

**Figure 15:**
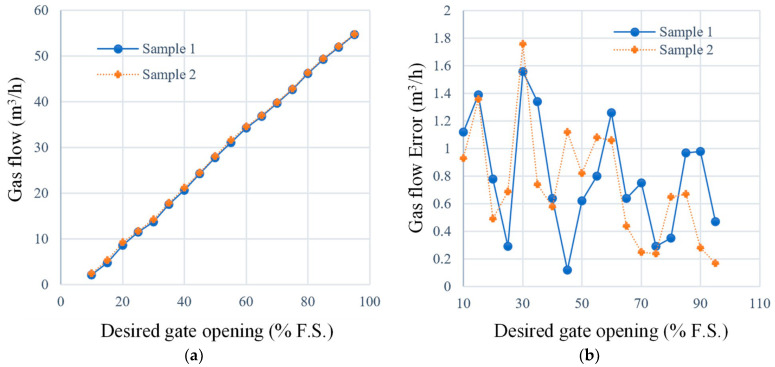
Gas flow control performance of EM valve. (**a**) Gas flow under different gate opening; (**b**) gas flow error to theoretical linear fitting value.

Figure 15 shows the flow characteristics of the different devices at various valve openings, and we find no obvious difference in error performance.

A strong linear relationship exists between the airflow and the valve opening, which indicates that the proposed method effectively suppresses both internal and external uncertainties and can accurately control the airflow at different valve openings.

**Figure 16:**
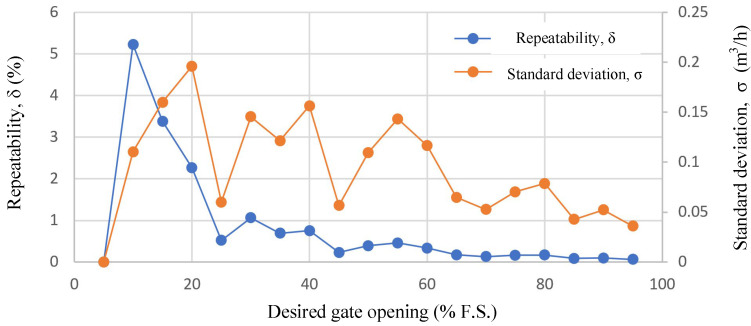
Repeatability of the gas flow control performance of the EM valve.

To verify the repeatability of the control performance of the system design. The repeatability of the airflow values was verified by varying the valve openings. The repeatability equation is given in Equations (33) and (34).

(33) $$\sigma =\sqrt{\frac{\sum {\left(x_{i}-X\right)}^{2}}{n-1}}$$

(34) $$\delta =\frac{\sigma }{X}\times 100\%$$

where *n* is the number of measurements, and *n* = 20. The overall airflow control repeatability of the servo system is shown in Figure 16. The repeatability of the airflow control is generally within 5%, and its standard deviation is less than 0.2 m^3^/h. From the error trend analysis, the repeatability and standard deviation of the airflow control at lower valve openings are inferior to those at larger valve openings. The reason for this is that the low valve opening region is also the strong cogging torque region of the LTAM, and its expected electromagnetic torque is small, resulting in a weak positional stiffness.

## 6. Conclusions

This paper proposes a design scheme for a LADRC-based position servo control system for electromotive valves for exhaust gas recirculation, which aims to address the problem of uncertainty disturbance in the practical application of electromotive EGR valves. Numerous simulation and experimental results demonstrate the following:

(1) This paper proposes a design scheme for a LADRC-based position servo control system that can effectively guarantee the performance of the electromotive valve under slowly varying plant dynamics and uncertain disturbances.
(2) The method presented in this paper exhibits good real-time response capability and strong suppression capability against external shock-type disturbances. The EM valve quickly returns to the desired position after the occurrence of a shock response, with a maximum deviation of 1.07 mm. In addition, the steady-state error after recovery is less than 0.001 mm, and the recovery time after the shock-type disturbance is about 240 ms.
(3) The flow characteristics of different parts under different valve openings are very close to each other, and there is no noticeable difference in the error performance, and the airflow and the valve opening present a very close linear relationship, which indicates that the method in this paper has a good suppression effect on the internal and external uncertainties, and it can achieve accurate control of airflow at different valve openings.
(4) The system design has good performance in terms of airflow control. The repeatability of airflow control is generally within 5%, and its standard deviation is less than 0.2 m^3^/h.

Future work should take into account the following issues: (1) The presence of components such as springs in EGR valve applications and the dynamic process of switching between kinetic and static friction, resulting in strongly non-linear dynamics; and (2) the fact that the motion of the EGR valve is limited in stroke and characterized by reciprocity. Therefore, to improve the performance of the servo system, online system parameter identification or iterative control methods, such as iterative adjustment of control parameters for its full-motion stroke, can be used. This will be the next step in the research direction.

## Figures and Tables

**Figure 3:**
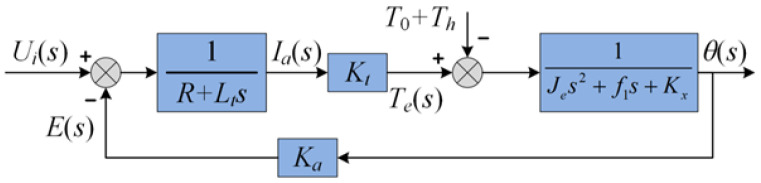
Block diagram of the LATM model.

**Table 1:** Parameters of the EM valve discussed in this paper.

Symbol	Parameter	Value
R	Equivalent resistance of armature winding	3.5 Ω
$L_{t}$	Winding inductance	32 mH
$V_{in}$	Input voltage of power stage	12 V
$K_{a}$	Back electromotive force coefficient	0.126 V⋅s/rad
$J_{e}$	Motor shaft moment of inertia	0.0019 $kg\cdot m^{2}$
$K_{x}$	Spring torque coefficient	0.051 N⋅m/rad
$f_{1}$	Motor shaft viscous damping coefficient	0.0025 N⋅m⋅s/rad
$k_{1}$	Proportional coefficient of transmission	0.0008 --
$k_{2}$	Proportional coefficient of transmission	0.0486 --
$T_{0}$	Torque spring initial torque	0.04 N⋅m
$f_{2}$	Equivalent viscous friction coefficient	0.013 N⋅m⋅s/rad

## Data Availability

Data are contained within the article.

## References

[1] Mourad M., Mahmoud K., Mohamed S. (2021). Improving diesel engine performance and emissions characteristics fuelled with biodiesel. Fuel.

[2] Jiang X., Wei H., Zhou L., Chen R. (2019). Numerical Study on the Effects of Multiple-Injection Coupled with EGR on Combustion and NOx Emissions in a Marine Diesel Engine. Energy Procedia.

[3] Reddy E., Keerthana B.V.S., Raju V.D., Sai M., Dhanush B. (2020). Mitigation of NOx emissions with application of exhaust gas recir-culation on diesel engine fuelled with diesel-corn seed oil biodiesel blend. Int. J. Ambient Energy.

[4] Sun C., Liu Y., Qiao X., Ju D., Tang Q., Fang X., Zhou F. (2020). Experimental study of effects of exhaust gas recirculation on combustion, performance, and emissions of DME-biodiesel fueled engine. Energy.

[5] Lu D., Theotokatos G., Zhang J., Zeng H., Cui K. (2022). Comparative Assessment and Parametric Optimisation of Large Marine Two-Stroke Engines with Exhaust Gas Recirculation and Alternative Turbocharging Systems. J. Mar. Sci. Eng..

[6] Nag S., Sharma P., Gupta A., Dhar A. (2019). Experimental study of engine performance and emissions for hydrogen diesel dual fuel engine with exhaust gas recirculation. Int. J. Hydrogen Energy.

[7] Wu H.W., Hsu T.T., He J.Y., Fan C.M. (2017). Optimal performance and emissions of diesel/hydrogen-rich gas engine varying intake air temperature and EGR ratio. Appl. Therm. Eng..

[8] Öztürk E., Can Ö. (2022). Effects of EGR, injection retardation and ethanol addition on combustion, performance and emissions of a DI diesel engine fueled with canola biodiesel/diesel fuel blend. Energy.

[9] Aravind M.A., Dinesh N.S., Rajanna K. (2020). Application of empc for precise position control of dc-motor system with backlash. Control Eng. Pract..

[10] Altan A., Hacioglu R. (2020). Model predictive control of three-axis gimbal system mounted on UAV for real-time target tracking under external disturbances. Mech. Syst. Signal Process..

[11] Kumar S.S., Anitha G. (2021). A novel self-tuning fuzzy logic-based PID controllers for two-axis gimbal stabilization in a missile seeker. Int. J. Aerosp. Eng..

[12] Lee D.H., Tran D.Q., Kim Y.B., Chakir S. (2020). A robust double active control system design for disturbance suppression of a two-Axis gimbal system. Electronics.

[13] Shen T., Kang M., Gao J., Zhang J., Wu Y. (2018). Challenges and solutions in automotive powertrain systems. J. Control Decis..

[14] Oltean S.E., Dulau M., Duka A.V. (2016). Model reference adaptive control design for slow processes. A case study on level process control. Proc. Technol..

[15] Lu J., Chang S. (2019). Precise motion control of an electromagnetic valve actuator with adaptive robust compensation of combustion force. J. Frankl. Inst..

[16] Choi S.Y., Lee S.H., Lee S.B. (2014). A study on the electronic EGR valve control method. J. Korea Acad. Ind. Coop. Soc..

[17] Kim H.J., Son Y.D., Kim J.M. (2020). Improved Position Control for an EGR Valve System with Low Control for an EGR Valve System with Low Control Frequency. Energies.

[18] Bhuiyan H., Lee J.-H. (2018). Low cost position controller for exhaust gas recirculation valve system. Energies.

[19] Ashok B., Denis A.S., Ramesh K.C. (2017). An Integrated Pedal Follower and Torque Based Approach for Electronic Throttle Control in a Motorcycle Engine. Eng. J..

[20] Sheng W., Bao Y. (2013). Fruit fly optimization algorithm based fractional order fuzzy-PID controller for electronic throttle. Nonlinear Dyn..

[21] Rajaei N., Han X., Chen X., Zheng M. (2010). Model Predictive Control of Exhaust Gas Recirculation Valve.

[22] Cheng W., Tan Y., Lu Y., Dong R., Tan Q., Chen X. Nonlinear internal model control of EGR valve. Proceedings of the 2019 12th Asian Control Conference (ASCC).

[23] Tan Y., Cheng W., Dong R., Tan Q., Cao Q. (2020). Online optimizing positioning control with model error compensator for LEGRV system. IEEE/ASME Trans. Mechatron..

[24] Rajaei N. (2010). Model Based Control of Exhaust Gas Recirculation Valves and Estimation of Spring Torque. Master’s Thesis.

[25] Gao J., Feng K., Wang Y., Wu Y., Chen H. (2020). Design, implementation and experimental verification of a compensator-based triple-step model reference controller for an automotive electronic throttle. Control Eng. Pract..

[26] Li Y., Yang B., Zheng T., Peeta S. (2015). Extended-State-Observer-Based Double-Loop Integral Sliding Mode Control of Electronic Throttle Valve. IEEE Trans. Intell. Transp. Syst..

[27] Liu J., Zhao Y., Fan X., Zhang X. (2019). The Invention Relates to a Stator Double Excitation Torque Motor with Limited Turning Angle. Micromotors.

[28] Guo L., Zheng C.L., Wang H. (2021). Design and Simulation of Semi-Immersed Limited Angle Torque Motor. Small Spec. Electr. Mach..

[29] Yu G., Xu Y., Zou J., Xiao L., Zheng B. (2020). Modeling and analysis of limited-angle torque motor considering nonlinear effects. IEEE Trans. Transp. Electr..

[30] Yang Y., Wang J., Zhou S., Huang T. (2019). Design of a novel coaxial eccentric indexing cam mechanism. Mech. Mach. Theory.

[31] Han J. (2009). From PID to active disturbance rejection control. IEEE Trans. Ind. Electron..

[32] Wang Z., Gong Z., Chen Y., Sun M., Xu J. (2020). Practical control implementation of tri-tiltRotor flying wing unmanned aerial vehicles based upon active disturbance rejection control. Proc. Inst. Mech. Eng. Part G J. Aerosp. Eng..

[33] Fu T., Gao Y., Guan L., Qin C. (2022). An LADRC Controller to Improve the Robustness of the Visual Tracking and Inertial Stabilized System in Luminance Variation Conditions. Actuators.

[34] Gao Z. Scaling and bandwidth-parameterization based controller tuning. Proceedings of the 2003 American Control Conference.

